# A Practical Treatise on Pulmonary Consumption, Its Pathology, Diagnosis, and Treatment, &c.

**Published:** 1843-04

**Authors:** 


Art. VII.—1. *A Practical Treatise on Pulmonary Consumption, its Pathology, Diagnosis, and Treatment, &c.* By Francis Cook, m.d. m.r.c.s.e.—*London*, 1842. 8vo, pp. 118.

2. *An Exposition of the Pathology and Treatment of Tubercular Phthisis.* By Samuel Flood, m.r.c.s.—*London*, 1842. 12mo, pp. 82.

We had recently occasion to notice in terms of censure, a work professedly descriptive of consumption; and we have now evidence before us that the desire of acquiring a small notoriety in connexion with that disease, possesses more than one person whose qualifications scarcely average those of moderately intelligent students. Where this sudden passion for tacking together worn-out platitudes, or painfully absurd novelties on the subject of phthisis, is to end, we cannot pretend to determine; and indeed judging from late experience, we may presume that a volume on consumption may be looked forward to by those interested in the progress of the exact sciences, as an event destined to occur quarterly.

But this matter grows too serious to be treated triflingly. For, though we are unwilling to acquiesce in the common notion that the learned writers produce these books with the simple view of advertising themselves, and for our own parts feel contented to regard them as the offspring solely of a somewhat impertinent vanity, yet productions of the sort are not as harmless as they might appear. They in truth lend their little aid in supporting and giving vigour to the monstrous system of fraud practised by the "consumption-curing" doctors on the too gullible multitude. Viewed in this aspect they are mischievous in the extreme; though redolent of the very refuse of Paternoster Row, their influence upon the sufferers from consumption will not be the less active. Though utterly destitute of logic, as of evidence of even moderate acquaintance on the part of the writers with the principles of precise medicine, they will not fail, because they hold out hopes of cure, to lure many a victim to the toils set for all whose pockets are sufficiently well lined to make them worth the catching.

With these general observations we dismiss the two works now before us; we will not waste the time of our readers or our own, in attempting to expose their inanities. They contain not a single novelty of the least conceivable value; but they abound with old errors, and silly suggestions both old and new.

